# Reliability and Intra Urban Variability of a Quality of Life Index: Findings of Pilot Studies of Young Women in Kampala, Uganda

**DOI:** 10.24248/eahrj.v9i2.859

**Published:** 2025-12-24

**Authors:** Rachel E. Culbreth, Monica H. Swahn, Jane Palmier, Rogers Kasirye

**Affiliations:** a American College of Medical Toxicology, 10645 N. Tatum Blvd, Phoenix, Arizona 85028; b School of Public Health, Virginia Commonwealth University, Richmond, VA 23219, USA; c Uganda Youth Development Link, Kampala, Uganda

## Abstract

**Background:**

Research on health and quality of life among young adults living in the slums is relatively sparse. This analysis aimed to: 1) assess the reliability of the Uganda Youth Quality of Life (QOL) Index across two pilot studies, and 2) examine intra-urban differences in QOL domains among young women in three slum communities of Kampala.

**Methods:**

Data from two consecutive pilot studies conducted among young women and girls in Kampala, Uganda were utilized for this analysis (n=60). These pilot studies were originally designed to examine the feasibility and acceptability of daily diaries and wearable devices for the larger cohort study (“TOPOWA”, meaning empowerment). The QOL domains (living conditions and lifestyle, social relationships, and personal independence) were obtained from the Uganda Youth Quality of Life Index (part of baseline survey). Comparisons in QOL domains between the two pilots were conducted to examine reliability, and inter-urban differences were analyzed across the three study sites (Banda, Bwaise, and Makindye).

**Results:**

The mean age of all participants was 20.1 years, and nearly one-third reported having children. While the QOL index demonstrated reliability across pilots (no significant differences in domain scores or life satisfaction, p=0.59), significant intra-urban differences were found. Participants in Bwaise reported consistently higher satisfaction across multiple domains compared to Banda and Makindye. However, satisfaction with their current dwelling was highest in Makindye (F=4.7, *p*=.02). Overall life satisfaction also differed significantly by site (F=5.2, *p*=.008).

**Conclusions:**

The Uganda Youth Quality of Life Index was reliable across the two pilot studies. However, intra-urban differences were noted across Kampala, suggesting that urban slum communities have a high degree of variability in life satisfaction and may warrant targeted interventions which address community differences.

## BACKGROUND

There has been a surge of literature defining and examining the urban health context, including health risks and behaviors within urban slums. Urban slums present complex and multifaceted challenges, particularly in developing countries. Slums typically consist of informal settlements, often characterized by overcrowding, poor sanitation, poverty, dire environmental living conditions, and social inequities.^1^ With over 60% of sub-Saharan Africa's urban population living in slum conditions, it is evident that this is a significant concern.^2^ The number of slum dwellers is expected to grow globally, reaching over 1 billion people currently living in slums, further exacerbating the issue.^3^

Urban slums present significant public health challenges, particularly in low-income countries. Studies have shown that urban slum residents face worse health outcomes compared to other urban populations and even rural counterparts.^4,5^ While there has been a growing interest in urban health, research on health and QOL among those living in slums remains relatively sparse. This is likely the case because typical research methodologies rely on clinical samples and health systems for accessing populations. Those living in slums are often marginalized with less access to health care and other infrastructure so are often considered “hard to reach” in typical research studies.^6^

People living in slum communities face a myriad of social, economic, and environmental challenges. These communities are susceptible to climate disasters and the resulting coping strategies have been investigated, highlighting the need for resiliencebuilding measures in these areas.^7^ Socioeconomic challenges include low literacy, poor housing, and limited access to education and employment opportunities.^3,8^ Protective factors such as resilience in slum communities have also been explored, with findings indicating that social capital and communityled initiatives play a crucial role in enhancing protecting slum community members from threats such as eviction and environmental hazards.^9,10^

Multiple factors influence QOL for slum dwelling communities, emphasizing the need for a validated and holistic QOL index. Previous research^6,11–14^ delve into the prevalence of HIV, alcohol use, problem drinking, violence, and risky behaviors among youth in the slums of Kampala, who are a particularly vulnerable population. Youth living in the slums of Kampala face high rates of sexual victimization, adolescent pregnancies, intimate partner violence, and psychological distress,^(12),12–25^ emphasizing the urgent need for interventions and prevention strategies. However, quantifying the impact and burden on QOL is difficult without a validated measure needed to inform health programs and initiatives.

Despite the context of the challenges faced by those living in slums, and the clear impact on their QOL, few studies have sought to assess the QOL as a key measure in these populations in low-resource settings.^26,27^ The integrative quality-of-life (IQOL) theory provides a comprehensive framework for measuring the global QOL, encompassing aspects such as well-being, satisfaction with life, happiness, and meaning in life.^28^ This highlights the need for a holistic approach to QOL assessment that includes many components and allows for the assessment of their interrelations.^29^ This was the approach taken by the team developing the Ugandan Youth Quality of Life index.^30^ This index was designed to factor in the perceived importance of the QOL measure and identifies factors associated with the QOL among youth in slum areas of Kampala.^30^ Moreover, the relationship between skills development support and self-reliance among youth in Lira City, Uganda, underscores the significance of socio-economic factors in shaping the QOL of Ugandan youth.^31^ This QOL index also showed improvement in the living conditions domain for participants who received an intervention titled, “Urban Program on Livelihoods and Income Fortification and Socio-civic Transformation (UPLIFT)”.^32^

To prepare for a prospective cohort study on the social drivers of mental illness of young women residing in three different slums in Kampala, we included the Uganda Youth QOL Index in two consecutive pilot studies. Although the primary aim of the pilots was to test wearable devices for measuring sleep and activity patterns, the concurrent administration of the Uganda Youth QOL Index provided a unique opportunity to evaluate the psychometric properties and contextual variability of this key metric in our target population prior to the main cohort study. The QOL index was included to understand the demographic and contextual factors related to the social drivers for the young women living in the urban slums. This analysis sought to examine preliminary data on QOL domains (i.e., living conditions and lifestyle; social relationships; and personal independence) to ultimately inform the relevant measures for cohort study inclusion. Comparisons in QOL domains between the two pilot studies were conducted to examine variability and reliability of this QOL index in the target population. Finally, we wanted to know if there were intra-urban differences in the quality of life across the three study sites within Kampala. These research questions are important for our ongoing research on the social and contextual factors for young women living in the urban slums and presented a unique opportunity to assess some key methodological issues related to assessing QOL in this population which may inform other epidemiological research, prevention and intervention programs in similar settings.

## METHODS

### Participant Recruitment

This study consists of data obtained from two consecutive pilot studies designed to examine the feasibility and acceptability of using daily diaries and wearable sleep and activity trackers for young women living in the slums of Kampala. These two pilot studies were conducted to inform the implementation of a larger longitudinal cohort study called TOPOWA (meaning empowerment) of young women. The first pilot study (“Pilot 1”) recruited participants between January 23 to 25, 2023, and the second pilot study (“Pilot 2”) recruited participants between May 3 to 4, 2023.

The broader NIH-funded TOPOWA project is comprised of several qualitative studies including focus groups and a photovoice project^33^ as well as the main TOPOWA cohort study, all designed with a shared goal of understanding and mitigating the social determinants of mental health among adolescent girls and young women living in the slums of Kampala.^34^

For the current TOPOWA project pilot studies, a total of 60 women (n=30 from each pilot study) ages 18 to 24 were recruited from three neighborhoods in Kampala: Banda, Bwaise, and Makindye. Ten women from each neighborhood who were seeking services at Uganda Youth Development Link (UYDEL) were recruited for each pilot study (in spring of 2023), resulting in 20 women total from each site across the two pilot studies. Participants were asked to complete a short survey as part of the pilot study.

### Patient and Public Involvement Statement

Young women living in the slums of Kampala participated in the community/participant advisory board, which gave feedback on specific measures used in the pilot studies, cohort surveys and design of training materials. While these young women were not directly involved in the research question and outcome development, the community advisory board was instrumental in developing survey guidelines and instructions and protocol development.

### Measures

The Ugandan Youth QOL Index was used to measure life satisfaction on domains such as friendships, family relationships, money and financial security, neighborhood cohesion, and access to health and transportation service.^30^ The Ugandan Youth QOL Index was operationalized as the mean satisfaction score (1–5 with 5 being very satisfied and 1 being very unsatisfied) for each of the three domains: Living conditions and lifestyle, social relationships, and personal independence. The overall life satisfaction was measured using a ladder scale for a total of 10 possible response options with 1 being the worst possible life satisfaction and 10 being the best possible life satisfaction.

The Ugandan Youth QOL Index is intended to be utilized with an importance rating scale.^30^ In line with the pilot nature of this analysis and the limited sample size, we utilized only the satisfaction components of the index to assess basic reliability and variability. The importance ratings will be incorporated in the larger cohort study. In the larger TOPOWA cohort study, the Ugandan Youth QOL Index will be operationalized using both the life satisfaction domains and the importance scales.

### Data Analysis

Descriptive statistics were computed for the Ugandan QOL Index for Pilot 1 and Pilot 2 by subscale, life satisfaction and study site location. Mean scores for each question and domain were compared using Independent Samples T-Tests for bivariate comparisons across pilot studies. Demographic characteristics were compared using Chi-Square Tests for categorical variables and Fisher's Exact Tests for expected cell counts <5. Differences across the three-study site locations were compared using one-way analysis of variance (ANOVA) tests. Missing data were examined and handled using listwise deletion for data when missingness was <10% of total sample. Statistical significance was set a priori at *P*=.05.

### Ethical Considerations

Ethical approvals were obtained from Kennesaw State University, the Makerere University School of Social Sciences Research Ethics Committee (MAKSSREC 06.22.566) and the Uganda National Council of Science and Technology (registration number SS1347ES). This study is compliant with the Declaration of Helsinki. Written informed consent was obtained from all participants. Participants were compensated for their time and completion of surveys. In these analyses we use the findings from the surveys across the two pilot studies.

## RESULTS

Demographic characteristics across both pilot studies are presented in Table 1. There were no differences in demographic characteristics between the two pilot studies. The mean age of all participants was 20.1 years (standard deviation=1.9 years; range 18–24). Among study participants, 33% reported having children, and 65% of mothers reported having only one child. None of the study participants lived by themselves; 43% of participants reported having four to six people living in their household. Half of participants completed some secondary schools, with 15% completing some primary school, and 15% fully completing primary school.

**TABLE 1:** Demographic Characteristics among TOPOWA Project Pilot Study Participants (N=60)

	First Pilot N=30	Second Pilot N=30	Total N=60	Statistical Test
Age, Mean (SD)	20.1 (2.0)	20.1 (1.7)	20.1 (1.9)	T=0.07, df=56.1, *p*=.94
Do you have children?				
Yes	9 (30.0%)	11 (36.7%)	20 (33.3%)	χ^2=0.1, df=1, *p*=1.00
No	21 (70.0%)	19 (63.3%)	40 (66.7%)	
If yes, how many children do you have?				
One	6 (66.6%)	7 (63.6%)	13 (65.0%)	*p*=.70^*^
Two	3 (33.3%)	2 (18.2%)	5 (25.0%)	
Three	0	2 (18.2%)	2 (10.0%)	
How many people are living in your household?				
One	0	0	0	*p*=.40^*^
Two	4 (13.3%)	8 (26.7%)	12 (20.0%)	
Three	7 (23.3%)	5 (16.7%)	12 (20.0%)	
Four to six	13 (43.3%)	13 (43.3%)	26 (43.3%)	
Seven to nine	4 (13.3%)	3 (10.0%)	7 (11.7%)	
Ten or more	2 (6.7%)	1 (3.3%)	3 (5.0%)	
Highest level of education				
Completed some primary school	3 (10.0%)	6 (20.0%)	9 (15.0%)	*p*=.09^*^
Completed primary school	3 (10.0%)	6 (20.0%)	9 (15.0%)	
Completed some secondary school	14 (46.7%)	16 (53.3%)	30 (50.0%)	
Completed secondary school	9 (30.0%)	2 (6.7%)	11 (18.3%)	
Completed tertiary school	1 (3.3%)	0	1 (1.7%)	

Note. Statistical tests are displayed using test statistic, degrees of freedom (df), and p-value. Independent samples T-Tests were used for age, chi-square test and Fisher's Exact Tests were used for categorical variables.

* Fisher's Exact Test used for expected cell counts <5.

Overall, the mean QOL index mean scores for Pilot 1 and Pilot 2, separate and in aggregate, are presented in Table 2. There were no significant differences across the three sub-scale domains (living conditions and lifestyle, social relationships and personal independence). Only one question was statistically significantly different across the pilot studies. Satisfaction with sex life was statistically significantly different across the two measures, with the first pilot resulting in higher satisfaction (mean score= 3.6, standard deviation= 1.0) compared to the second pilot (mean score= 2.7, standard deviation= 1.0; T=3.5, df=54.0, *p*<.001). This question also had 4 responses missing.

**TABLE 2:** Quality of Life Index Among Young Women Living in the Slums of Kampala, Uganda Across Two TOPOWA Project Pilot Studies, (N=60)

Subscale & Variables	First Pilot N=30	Second Pilot N=30	Total N=60	Statistical Test
**Living conditions & lifestyle domain**	2.7 (0.6)	2.6 (0.5)	2.6 (0.6)	T=0.57, (54.0), *p* =.57
How satisfied are you with the clothing you wear?	3.1 (1.1)	3.1 (1.0)	3.1 (1.0)	T=0, (57.9), *p* =1.00
How satisfied are you with the food you eat?	3.0 (1.2)	3.1 (1.0)	3.0 (1.1)	T=-0.2, (55.7), *p* =.81
How satisfied are you with your sex life?	3.6 (1.0)	2.7 (1.0)	3.2 (1.1)	T=3.5, (54.0), *p* <.001
How satisfied are you with your personal safety?	3.6 (1.0)	3.5 (0.9)	3.6 (1.0)	T=0.1, (56.8), *p* =.89
How satisfied are you with the health services you use?	2.9 (1.3)	3.0 (1.1)	3.0 (1.2)	T=-0.22, (56.4), *p* =.83
How satisfied are you with your access to transportation?	2.6 (1.2)	2.6 (1.0)	2.6 (1.1)	T=-0.1, (56.1), *p* =.90
How satisfied are you with the way you spend your time?	3.4 (1.1)	3.6 (0.8)	3.5 (0.9)	T=-0.54, (54.2), *p* =.59
**Social relationships**	3.3 (0.7)	3.1 (0.7)	3.2 (0.7)	T=1.5, (56.8), *p* =.14
How satisfied are you with how you get along with your friends?	3.5 (1.1)	3.1 (1.1)	3.3 (1.1)	T=1.6, (57.0), *p* =.12
How satisfied are you with the people with whom you live?	3.3 (1.3)	3.4 (1.2)	3.3 (1.2)	T=-0.29, (56.6), *p*=.78
How satisfied are you with the number of friends you have?	3.3 (0.93)	2.8 (1.1)	3.1 (1.0)	T=1.8, (56.2), *p*=.07
How satisfied are you with how you get along with other people in the community?	3.0 (1.2)	2.9 (1.1)	3.0 (1.2)	T=0.54, (57.5), *p*=.59
How satisfied are you with your neighborhood as a place to live in?^*^	3.0 (1.1)	2.9 (1.2)	3.0 (1.2)	T=0.33, (57.0), *p*=.74
How satisfied are you with your relationship with the other people in your family?	3.4 (1.0)	3.1 (1.2)	3.3 (1.1)	T=0.82, (55.2), *p* =.42
How satisfied are you with how you get along with your parent/guardian?	3.7 (1.0)	3.4 (1.3)	3.5 (1.1)	T=0.81, (52.5), *p* =.42
**Personal independence**	2.7 (0.7)	2.6 (0.8)	2.6 (0.7)	T=0.16, (54.6), *p* =.88
How satisfied are you with the amount of money you have?	2.3 (1.0)	2.3 (1.0)	2.3 (1.0)	T=0.10, (55.0), *p* =.92
How satisfied are you with the work opportunities available to you?	2.0 (0.8)	2.3 (1.1)	2.1 (1.0)	T=-1.1, (53.2), *p* =.28
How satisfied are you with the free time you have to comfortably be alone without any worry?	2.9 (1.0)	2.7 (1.1)	2.8 (1.0)	T=0.75, (55.3), *p*=.46
How satisfied are you with your current dwelling?	2.8 (1.1)	2.6 (1.1)	2.7 (1.1)	T=0.71, (55.9), *p* =.48
How satisfied are you with the amount of control you have over your own money?	3.4 (1.0)	3.3 (1.2)	3.3 (1.1)	T=0.24, (55.4), *p*=.81
Overall, how satisfied are you with your life, mean (SD) Range 1–10 (ladder)	4.1 (1.5)	4.3 (1.8)	4.2 (1.7)	T=-0.54, (55.8), *p*=.59

Note. Statistical tests are displayed using test statistic, degrees of freedom (df), and p-value. Independent samples T-Tests were calculated for differences in mean scores between pilot 1 and 2.

* =not included in the domain average of “Social Relationships” per original confirmatory factor analysis.

Lower mean satisfaction scores were observed for personal independence (mean score= 2.6, standard deviation= 0.7) and living conditions/lifestyle (mean score= 2.6, standard deviation= 0.6) compared to the social relationship's domain (mean score=3.2, standard deviation=0.7). The mean scores for “amount of money” (2.1) and “work opportunities” (2.8) were particularly low, indicating severe dissatisfaction in these economic aspects of personal independence.

In the comparison across the three study sites, the mean scores for all three subscale domains were significantly different. Bwaise consistently showed the highest satisfaction scores across the majority of items within the Living Conditions and Social Relationships domains. There were no statistically significant differences for several individual questions across the three study sites, including satisfaction with sex life, personal safety, community satisfaction, and parental relationship satisfaction. Additionally, there were no statistically significant differences between satisfaction scores for work opportunities across all three study sites. While participants in Bwaise consistently scored higher for life satisfaction on most domains, participants in Makindye had higher satisfaction scores related to their current dwelling (F=4.7, df=2, *p*=.02). Of the seven variables within the living conditions domains, five were statistically significant and varied by site. Of the seven variables within social relationships, three were statistically significant and varied by study site (Table 3).

**TABLE 3:** Quality of Life Index among Young Women Living in TOPOWA Project Pilot Studies (N=60)

Subscale & Variables, M (SD)	Banda (N=19)	Bwaise (N=21)	Makindye (N=20)	Statistical Test
**Living conditions and lifestyle domain**	2.0 (0.3)	3.0 (0.4)	2.7 (0.4)	F=33.7, (2), *p*<.001
How satisfied are you with the clothing you wear?	2.2 (0.6)	3.8 (0.7)	3.2 (1.1)	F=29.6, (2), *p*<.001
How satisfied are you with the food you eat?	2.2 (0.9)	3.5 (0.9)	3.3 (1.0)	F=10.9, (2), *p*<.001
How satisfied are you with your sex life? (missing N=4)	2.9 (1.2)	3.1 (1.2)	3.5 (0.9)	F=1.4, (2), *p*=.26
How satisfied are you with your personal safety?	3.6 (0.8)	3.9 (1.0)	3.2 (1.0)	F=2.5, (2), *p*=.09
How satisfied are you with the health services you use?	2.1 (0.8)	3.5 (1.1)	3.3 (1.1)	F=11.8, (2), *p*<.001
How satisfied are you with your access to transportation?	1.9 (0.6)	3.4 (1.1)	2.4 (0.8)	F=14.2, (2), *p*<.001
How satisfied are you with the way you spend your time?	2.8 (1.1)	4.0 (0.6)	3.7 (0.7)	F=8.5, (2), *p*<.001
**Social relationships**	2.6 (0.5)	3.5 (0.4)	3.5 (0.7)	F=18.8, (2), *p*<.001
How satisfied are you with how you get along with your friends?	2.4 (1.0)	3.7 (1.0)	3.7 (0.8)	F=12.1, (2), *p*<.001
How satisfied are you with the people with whom you live?	2.8 (1.1)	3.8 (1.0)	3.3 (1.3)	F=3.9, (2), *p*=.03
How satisfied are you with the number of friends you have?	2.4 (0.9)	3.5 (0.9)	3.3 (1.0)	F=8.5, (2), *p*<.001
How satisfied are you with how you get along with other people in the community?	2.6 (1.2)	3.0 (1.2)	3.3 (1.2)	F=1.9, (2), *p*=.17
How satisfied are you with your neighborhood as a place to live in?^*^	2.7 (1.2)	3.2 (1.2)	3.1 (1.1)	F=1.0, (2), *p*=.37
How satisfied are you with your relationship with the other people in your family?	2.6 (1.1)	3.6 (0.9)	3.5 (1.1)	F=5.1, (2), *p*=.009
How satisfied are you with how you get along with your parent/guardian?	3.2 (1.1)	3.9 (1.1)	3.6 (1.2)	F=1.9, (2), *p*=.16
**Personal independence**	2.4 (0.3)	3.2 (0.8)	2.3 (0.5)	F=8.8, (2), *p*<.001
How satisfied are you with the amount of money you have?	1.8 (0.4)	2.9 (1.2)	2.1 (1.0)	F=6.7, (2), *p*=.004
How satisfied are you with the work opportunities available to you?	1.9 (0.6)	2.6 (1.4)	1.9 (0.3)	F=2.0, (2), *p*=.16
How satisfied are you with the free time you have to comfortably be alone without any worry?	2.3 (0.7)	3.7 (0.9)	2.2 (0.7)	F=21.1, (2), *p*<.001
How satisfied are you with your current dwelling?	2.2 (0.8)	2.8 (1.3)	3.0 (1.0)	F=4.7, (2), *p*=.02
How satisfied are you with the amount of control you have over your own money?	3.7 (0.7)	3.9 (0.9)	2.4 (1.0)	F=15.2, (2), *p*<.001
Overall, how satisfied are you with your life, mean (SD) Range 1–10 (ladder)	3.4 (1.4)	5.0 (2.8)	4.2 (1.4)	F=5.2, (2), *p*=.008

Note. Statistical tests are displayed using test statistic, degrees of freedom (df), and p-value. One-way ANOVA tests were conducted for differences in means across the three study sites.

* =not included in the domain average of “Social Relationships” per original confirmatory factor analysis.

Overall, the mean life satisfaction scores were statistically significantly different across the three study sties (F=5.2, df=2, *p*=.008), but no differences were detected across the two pilot studies (T=-0.54, df=55.8, *p*=.59).

## DISCUSSION

The objective of this analysis was to examine the reliability and variability in QOL across the two pilot studies and three different study sites. The objective was to also determine which aspect of QOL within this established and validated Uganda QOL index (i.e., living conditions and lifestyle; social relationships; and personal independence) was most impactful for young women (meaning having the lowest score) living in urban Kampala. Overall, the findings across the two TOPOWA project pilot studies support the Ugandan QOL Index as a reliable measure of QOL when tested across two distinct populations with similar characteristics. The only measure that had statistically significant variability was the question on satisfaction with sex life. This question is likely to be affected by social desirability bias and may be perceived as a sensitive topic. Four total responses were missing from this summary score, which may also have affected the statistical significance. In our larger TOPOWA prospective cohort study, we plan to incorporate extensive training for research associates for asking sensitive questions on sexual risk behaviors. Furthermore, with the larger sample of 300 women in the TOPOWA cohort study, precision is likely to be improved in understanding this specific measure.

Taken as a whole, the findings from the QOL index show grave dissatisfaction with the quality of life across several key domains for the young women living in three urban slums in Kampala. The lowest satisfaction was observed for the subscale domain labeled personal independence. Within that domain, the lowest satisfaction was in terms of work opportunities and regarding the participants’ total amount of money. These findings are not unexpected given that they live in poverty and in harsh environments with limited opportunities and resources.^17,33,34^ However, the findings related to personal independence varied by study site and not across pilot studies.

There were other significant variations in QOL domain scores and individual satisfaction items across study sites. Overall, participants from Bwaise reported significantly higher scores in most life satisfaction and social relationship domains, whereas participants in Makindye reported significantly higher satisfaction scores with their current dwelling. These patterns may reflect differences in community cohesion, social networks and access to informal support systems, which are known to influence perceived well-being in low-resource settings. Bwaise, which is a more densely populated and longestablished settlement, may offer stronger communal support structures, informal employment opportunities and peer networks. And, these, collectively, may foster higher perceived quality of life across social and relational domains. In contrast, participants in Makindye reported lower scores in most domains may have comparatively better housing structures or more stable dwelling arrangements, contributing in turn to higher satisfaction with living conditions despite broader challenges.

Although systematic research comparing infrastructure and socioeconomic variation across Kampala's informal settlements is sparse, these preliminary findings underscore the heterogeneity of lived experiences across slum communities. They suggest that even within geographically proximate settings, differences in social capital, informal economies, service access, and tenure security may meaningfully shape quality-of-life perceptions. Understanding these intra-urban disparities is essential to designing context-sensitive and locationtailored interventions. Future analyses within the TOPOWA cohort study (N=300) will further explore these site based differences to inform targeted and communityspecific intervention approaches.

In contrast, satisfaction with work and finances showed no significant variation by site, likely reflecting uniformly limited employment opportunities across all three informal settlements. Young women in urban slum settings commonly rely on informal, unstable, and lowpaying work with few pathways for financial stability or upward mobility. These structural constraints may explain why work-related satisfaction was consistently low and not site-specific, suggesting that economic insecurity is a pervasive challenge across settlements, regardless of differences in housing or social cohesion.

Finally, for the overall life satisfaction measure, it had significantly different mean scores across the three study sites but not across the two pilot studies, indicating reliability and consistency in this measure across similar populations. The differences in the three study sites might be attributed to variations in slum characteristics; however, more research is needed to identify and understand these differences. These issues are important to factor in for future studies seeking to understand quality of life across potentially heterogeneous urban contexts and addressing methodological challenges when designing study or program evaluation protocols. Additionally, future studies should examine various populations and health outcomes with relation to this measure, including populations experiencing violence and substance use.

### Limitations

There are a few key limitations that need to be considered when interpreting the findings from this study. First, this study included analyses from two consecutive pilot studies which were originally intended to inform the feasibility and acceptability of the prospective cohort study component of the TOPOWA Project. Second, the overall sample size from this study is small, even when aggregating samples from Pilot 1 and Pilot 2, which means the findings may be subjected to insufficient statistical power or type I error rates for multiple statistical tests. Accordingly, participants may also not be representative of all women living in the slums of Kampala since these women were recruited through Uganda Youth Development Link Centers. Survey responses may be affected by social desirability and recall bias. However, the objective of this study was to assess methodological considerations for the implementation of the Uganda QOL index in populations living in urban slums. Finally, because of the small sample, some additional and specific analysis were not feasible, including the evaluation of the importance measures that are part of the QOL index. Despite the limitations, we found this QOL index of great value for our research and for contextualizing the key domains of life satisfaction for the young women who live in the urban slums of Kampala.

## CONCLUSIONS

Our findings offer several key methodological considerations for future research: (1) The Uganda Youth QOL Index is a reliable tool for this population, supporting its use in longitudinal studies; (2) Significant intra-urban heterogeneity necessitates either targeted sampling or sufficient sample size to account for cluster effects by neighborhood; and (3) Economic domains (money, work) are critical drivers of poor QOL in this context and must be included in assessments and addressed by interventions. Future research should also consider qualitative data to support the Uganda Youth QOL Index, which may inform policy decisions and targeted interventions. The QOL Index was easy to administer (in the form of a tablet administered survey) and to score. We found that the QOL index yielded very important information about our study population and contextualized factors that can be addressed in future research and in interventions.
